# TIMP1 Indicates Poor Prognosis of Renal Cell Carcinoma and Accelerates Tumorigenesis *via* EMT Signaling Pathway

**DOI:** 10.3389/fgene.2022.648134

**Published:** 2022-02-25

**Authors:** Yi Shou, Yuenan Liu, Jiaju Xu, Jingchong Liu, Tianbo Xu, Junwei Tong, Lilong Liu, Yaxin Hou, Di Liu, Hongmei Yang, Gong Cheng, Xiaoping Zhang

**Affiliations:** ^1^ Department of Urology, Union Hospital, Tongji Medical College, Huazhong University of Science and Technology, Wuhan, China; ^2^ Institute of Urologic Surgery, Tongji Medical College, Huazhong University of Science and Technology, Wuhan, China; ^3^ Department of Pathogenic Biology, School of Basic Medicine, Huazhong University of Science and Technology, Wuhan, China

**Keywords:** TIMP1, renal cell carcinoma (RCC), tumorigenesis, biomarker, EMT-epithelial to mesenchymal transition

## Abstract

Renal cell carcinoma (RCC) is one of the most common malignancies in the urinary system. The mortality of advanced RCC remains high despite advances in systemic therapy of RCC. Considering the misdiagnosis of early-stage RCC, the identification of effective biomarkers is of great importance. Tissue inhibitor matrix metalloproteinase 1 (TIMP1), which belongs to TIMP gene family, is a natural inhibitor of the matrix metalloproteinases (MMPs). In this study, we found TIMP1 was significantly up-regulated in cell lines and RCC tissues. Kaplan-Meier analysis revealed that high expression of TIMP1 indicated a poor prognosis. Multivariate analysis further indicated that TIMP1 overexpression was an independent prognostic factor of RCC patients. Furthermore, knockdown of TIMP1 *in vitro* suppressed the proliferation, migration, and invasion of RCC cells, while upregulating TIMP1 accelerated the proliferation, migration, and invasion of RCC cells. In addition, we also found that TIMP1 prompted the progression of RCC via epithelial-to-mesenchymal transition (EMT) signaling pathway. In conclusion, the present results suggested that TIMP1 indicated poor prognosis of renal cell carcinoma and could serve as a potential diagnostic and prognostic biomarker for RCC.

## 1 Introduction

Renal cell carcinoma (RCC) is one of the cancer types that originated from the renal epithelium, which accounts for most cancer-related deaths (Hsieh et al., 2017). The main histological subtypes of RCCs are clear cell (cc) RCC (∼70% of RCCs), papillary (p) RCC (10%–15% of RCCs), and chromophobe (ch) RCC (∼5% of RCCs) (Morris and Latif, 2017). Cancer-specific survival rates at 5 years for the above three types of RCC are 68.9%, 87.4%, and 86.7%, respectively (Cheville et al., 2003; Cinque et al., 2021). The five-year survival rate of early-stage RCC reaches 71%–88%. Still, the survival rate at 5 years of RCC plummets to only 12% when metastasis occurs according to the latest study (Leibovich et al., 2010; Cinque et al., 2021). Therefore, it is necessary to identify practical biomarkers for the early diagnosis of RCC.

Tissue inhibitor matrix metalloproteinase (TIMP) family comprises four paralogous genes (TIMP1, TIMP2, TIMP3, TIMP4) (Brew and Nagase, 2010). TIMPs participate in more protease-independent biological functions including anti-apoptosis, anti-angiogenesis, cell cycle regulation, and differentiation activities in epithelial or blood-derived cells (Seo et al., 2003; Cruz-Munoz et al., 2006; Jung et al., 2006; Taube et al., 2006; Grunwald et al., 2019). TIMP1 is a major member of the TIMP family with a molecular weight of 23 KDa, which consists of a two-domain structure possessing metalloproteinase-inhibitory and cytokine-like signaling activities (Grunwald et al., 2019). Previous studies showed that high expression of TIMP1 in tissue or blood suggested poor outcomes in various cancers (Holten-Andersen et al., 1999; Wang et al., 2013; Jackson et al., 2017). Song revealed that TIMP1 promoted tumor progression and suppressed apoptosis via FAK-PI3K/AKT and MAPK pathway in colon cancer (Song et al., 2016). Gong found that higher levels of TIMP1 expression were associated with poor prognosis in triple-negative breast cancer (Cheng et al., 2016). Hemmerlein investigated the expression of matrix metalloproteinases and their inhibitors in medulloblastomas and their prognostic relevance (Ozen et al., 2004). In the field of RCC, Kugler’s study showed that the balance of MMP-2 and MMP-9 to TIMP-1 and TIMP-2 expression was an essential factor in the aggressiveness of RCC (Kugler et al., 1998). Kallakury pointed out that increased expression of TIMP1 correlated with poor prognostic variables in RCC (Kallakury et al., 2001). Hence, we aimed to explore the relationship between the expression of TIMP1 and clinicopathological factors in renal cancer. Furthermore, we investigated the functional roles of TIMP1 and the underlying biological signal pathway in renal cancer cells.

## 2 Materials and Methods

### 2.1 RCC Tissue Samples

A total of 59 pairs of tumor and adjacent normal tissues were obtained from the Department of Urology, Union Hospital, Tongji Medical College (Wuhan, China) between January 2018 and January 2019. The normal kidney tissues were obtained from 2 cm away from the edge of lesions. The clinical information of these samples was presented in Table 1. The samples were divided into two groups. The first group was immediately stored in liquid nitrogen for RNA and protein extraction. The second group was fixed in formalin and embedded in paraffin, then used for immunohistochemistry assays. Among these samples, we randomly selected 8 pairs of tissues for protein detection, 20 pairs of samples for quantitative real-time PCR and 3 pairs of samples for immunohistochemistry. No patients received anticancer therapy before surgery. All patients gave written informed consent before inclusion in this study, and the study was approved by the Human Research Ethics Committee of Huazhong University of Science and Technology. The study complies with the guidelines of the Declaration of Helsinki.

**TABLE 1:** Clinical characteristics of 59 patients with renal cell carcinoma.

Characteristic	Data
Age, mean ± SEM (years)	52.3 ± 13.8
Gender, male/female	31/28
Tumor size, mean ± SEM (cm)	5.5 ± 3.1
Location, right/left	27/32
T stage, n (%)	
T1a	13 (22.03)
T1b	28 (47.46)
T2a	8 (13.56)
T2b	5 (8.47)
T3	2 (3.39)
T4	2 (3.39)
Unknown	1 (1.69)
N stage, n (%)	
N0	54 (91.53)
N1	5 (8.47)
M stage, n (%)	
M0	56 (94.92)
M1	3 (5.08)
Fuhrman grade, n (%)	
1	14 (23.73)
2	27 (45.76)
3	9 (15.25)
4	4 (6.78)
Unknown	5 (8.47)

### 2.2 Cell Culture

The human renal proximal tubular epithelial cell line HK-2, and 5 kinds of human renal cell carcinoma cell lines: 786-O, ACHN, A498, CAKI-1, and OSRC-2, were used in this study and were obtained from the American Type Culture Collection. These cell lines were used for RT-qPCR and western blotting. The cells were grown in Dulbecco’s modified Eagles medium containing high glucose (4.5 g/L), fetal bovine serum (10%), and penicillin/streptomycin solution (1%). All cells were cultured under standard conditions: at 37°C in a 5% CO_2_ atmosphere.

### 2.3 Immunohistochemical Staining Assays

The paired RCC tissues and adjacent normal tissues were first fixed in 4% formalin at room temperature for 12 h, dehydrated, and embedded in paraffin. Then the tissue sections were incubated with rabbit TIMP1 monoclonal antibody (Abcam, ab109125, 1:1,000) overnight at 4°C. Tissue sections were washed three times with phosphate-buffered saline and incubated with secondary antibodies that were conjugated to horseradish peroxidase at room temperature for 2 h. The sections were scanned by a NanoZoomer S360 (Hamamastu Corporation) and observed with NDP.view2 software (Hamamastu Corporation). Random fields were selected to interpret the expression of TIMP1 in tissue sections under 100x and 400x magnification.

### 2.4 RNA Extraction and RT-qPCR

Total RNA was isolated from tissues or cells using TRIzol® reagent (Thermo Fisher Scientific, Inc.). The concentration and purity of the RNA solution were detected using a NanoDrop 2000 spectrophotometer (NanoDrop Technologies; Thermo Fisher Scientific, Inc.). Total RNA was then reverse transcribed into cDNA using a Superscript II reverse transcription kit (Takara Bio, Inc.) according to the manufacturer’s protocols. All the experiments were repeated thrice for all the samples. The primers used to amplify TIMP1 and GAPDH were synthesized by TSINGKE Inc. The sequences of forward and reverse primers were as follows: TIMP1-forward: 5′-CGC AGC GAG GAG GTT TCT CAT-3’; TIMP1-reverse: 5′-GGC AGT GAT GTG CAA ATT TCC-3’. GAPDH-forward: 5′-CGT GGA AGG ACT CAT GAC CA-3’; GAPDH -reverse: 5′-GCC ATC ACG CCA CAG TTT C-3’.

### 2.5 Western Blot Assays

Total protein was extracted from RCC tissues and corresponding adjacent normal tissues of 12 patients using RIPA lysis buffer (Servicbio.Inc.) with protease inhibitor phenyl methane sulfonyl fluoride (PMSF, 1%), and then the concentration was measured with BCA protein assay kit (Beyotime Biotechnology, Jiangsu, China). Primary rabbit polyclonal antibody against primary antibodies (TIMP1 1:1,000, Abcam,Inc., ab109125; N-cadherin 1:5000, Abcam.Inc., ab76011; E-cadherin 1:10000, Abcam,Inc., ab40772) and β-actin (1:10000; Abclonal,Inc., cat.AC026) were incubated overnight at 4°C. All the procedures were performed according to the manufacturer’s instructions.

### 2.6 Small Interfering RNA and Plasmids Construction, Transfections

Small interfering RNA (siRNA) oligonucleotide sequences specifically targeting TIMP1 (si-TIMP1) and negative control (si-NC) siRNA (cat. no. siBDM 1999A) were obtained from Guangzhou RiboBio Co., Ltd. The plasmids harboring TIMP1 (ov-TIMP1) and negative control (ov-NC) were constructed and supplied by Vigene Biology (Vigene, China). Cells were collected for subsequent experiments 48 h post transfection. The si-TIMP1 sequence was as follows: 5′-GCC AAT GTG ATG GTG GAC A-3'. The information about the plasmid that has been used for over expression of TIMP1 was provided in Supplementary Figure S1.

### 2.7 Cell Proliferation Assay

One thousand transfected cells were added to each hole in the 96-well plates. Cell proliferation was assessed by the CCK-8 assay (CCK8; Dojindo Molceular Technologies, Inc.) at 24, 48, 72, and 96 h following treatments, according to the manufacturer’s instructions. Ten μl of CCK8 was added to each hole and incubated with cells for 2 h. Then the optical density values were measured by a spectrophotometer at 450 nm to estimate the number of living cells.

### 2.8 Cell Migration and Invasion Assays

We planted 1 × 10^4^ cells into the upper chambers in serum-free medium for migration and 2 × 10^4^ cells for invasion. Sixty μl Matrigel (Thermo Fisher Scientific; Waltham, USA) had been added into the upper chambers for invasion assays. The lower chambers were filled with 600 μl DMEM added with 10% FBS. After 24 h of incubation, the cells were fixed in 100% methanol, then stained with 0.05% crystal violet. Finally, the results were observed under a light microscope at 100x magnification, and the cells passed through the membrane were counted in 3 randomly chosen fields.

### 2.9 Bioinformatics Analysis

TIMP1, TIMP2, TIMP3, and TIMP4 mRNA expression and clinical information of The Cancer Genome Atlas (TCGA) clear cell renal cell carcinoma dataset (TCGA_KIRC) were downloaded from the Xena Functional Genomics Explorer of University of California Santa Cruz (https://xenabrowser.net/). Beroukhim renal, Jones renal, and Yusenko renal datasets (Jones et al., 2005; Beroukhim et al., 2009; Yusenko et al., 2009) were obtained from the Oncomine database (https://www.oncomine.org). The gene set enrichment analysis (GSEA) platform with the Kyoto Encylopedia of Genes and Genomes and Gene Ontology databases (c2.all.v6.2.symbols.gmt) was employed to find pathways enriched in the gene set, based on the pathway Enrichment Score (ES). STRING (Version11.0) was used to explore the protein-protein reaction and biological function of TIMP1(https://string-db.org/).

### 2.10 Statistical Analysis

All statistical analyses were processed by GraphPad Prism 7.0 (GraphPad Software, Inc., USA) and SPSS Statistics 22.0 software (IBM SPSS, Chicago, IL, United States). Data of paired cases were analyzed using a paired student t-test, while analysis of unpaired cases was performed using a one-way analysis of variance (ANOVA) or t-test. Pearson’s χ2 test was applied to analyze the relationship between TIMP1 and TIMP3 expression and clinical parameters. The Kaplan-Meier analysis was used to estimate the correlation between TIMP1 and TIMP3 mRNA expression with overall survival (OS) and disease-free survival (DFS) times with the log-rank test. The TIMP1 mRNA levels downloaded from the TCGA_KIRC dataset were first divided into two groups according to different clinical parameters and then applied to draw receiver operating characteristic (ROC) curves and analyze the area under the curve (AUC) with GraphPad Prism 7.0. The diagnostic value of TIMP1 mRNA expression in RCC was evaluated by ROC curves and AUC. Finally, univariate and multivariate Cox proportional hazard regressions were applied to determine the prognostic significance of TIMP1 and TIMP3. All experiments were repeated thrice independently and all data were represented as mean ± SEM. A confidence threshold, *p* < 0.05, was considered to be statistically significant. *p < 0.05; **p < 0.01; ***p < 0.001; ****p < 0.0001.

## 3 Results

### 3.1 TIMP1 Was Upregulated in RCC

**FIGURE 1:**
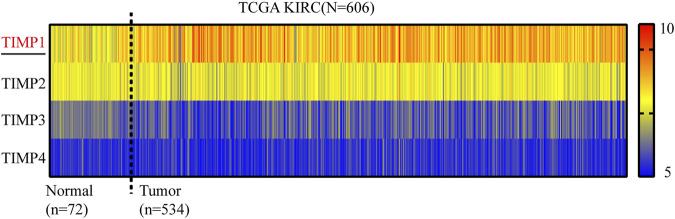
Heatmap of mRNA expression levels of TIMP family obtained from TCGA_KIRC. Red represented high expression and blue represented low expression. TIMP, Tissue inhibitor matrix metalloproteinase; KIRC, kidney renal clear cell carcinoma; TCGA, The Cancer Genome Atlas.

**FIGURE 2:**
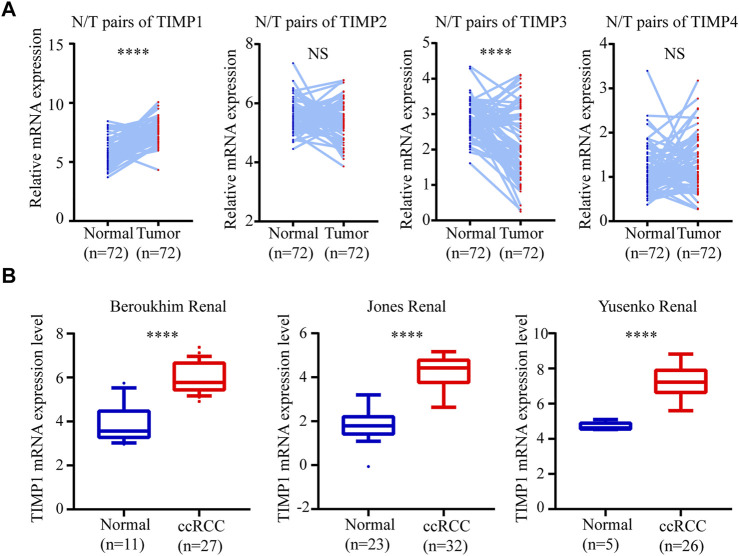
TIMP1 was upregulated in RCC. The mRNA expression level and clinical parameters were downloaded from TCGA_KIRC. **(A)** mRNA levels of TIMP family proteins in RCC tissues and paired normal tissues. **(B)** TIMP1 was upregulated in three renal statistics downloaded from the Oncomine database, including Beroukhim, Jones, and Yusenko renal statistics. TIMP, Tissue inhibitor matrix metalloproteinase; TIMP1, Tissue inhibitor matrix metalloproteinase 1; RCC, clear cell renal cell carcinoma, TCGA, The Cancer Genome Atlas; KIRC, kidney renal clear cell carcinoma.

To determine whether TIMP expression was related to the occurrence and progress of RCC, we downloaded the mRNA of 4 members in this family (TIMP1, TIMP2, TIMP3, and TIMP4) from TCGA_KIRC and drew a heatmap of them according to their mRNA expression. The result showed that TIMP1 was upregulated in RCC tissues (Figure 1). It was further verified that TIMP1 was found in higher expression levels in paired comparison (Figure 2A). To confirm these results, we compared the mRNA expression of TIMP1 in Oncomine datasets (Beroukhim, Jones, and Yusenko) and it was shown that TIMP1 was highly expressed in renal cancer samples (Figure 2B). All these results indicated that TIMP1 might play an important role in RCC progression, which raised our interest to further study.

### 3.2 High Level of TIMP1 Indicated a Poor Clinical Outcome in Subgroups of Patients With Different Clinical Parameters

**FIGURE 3:**
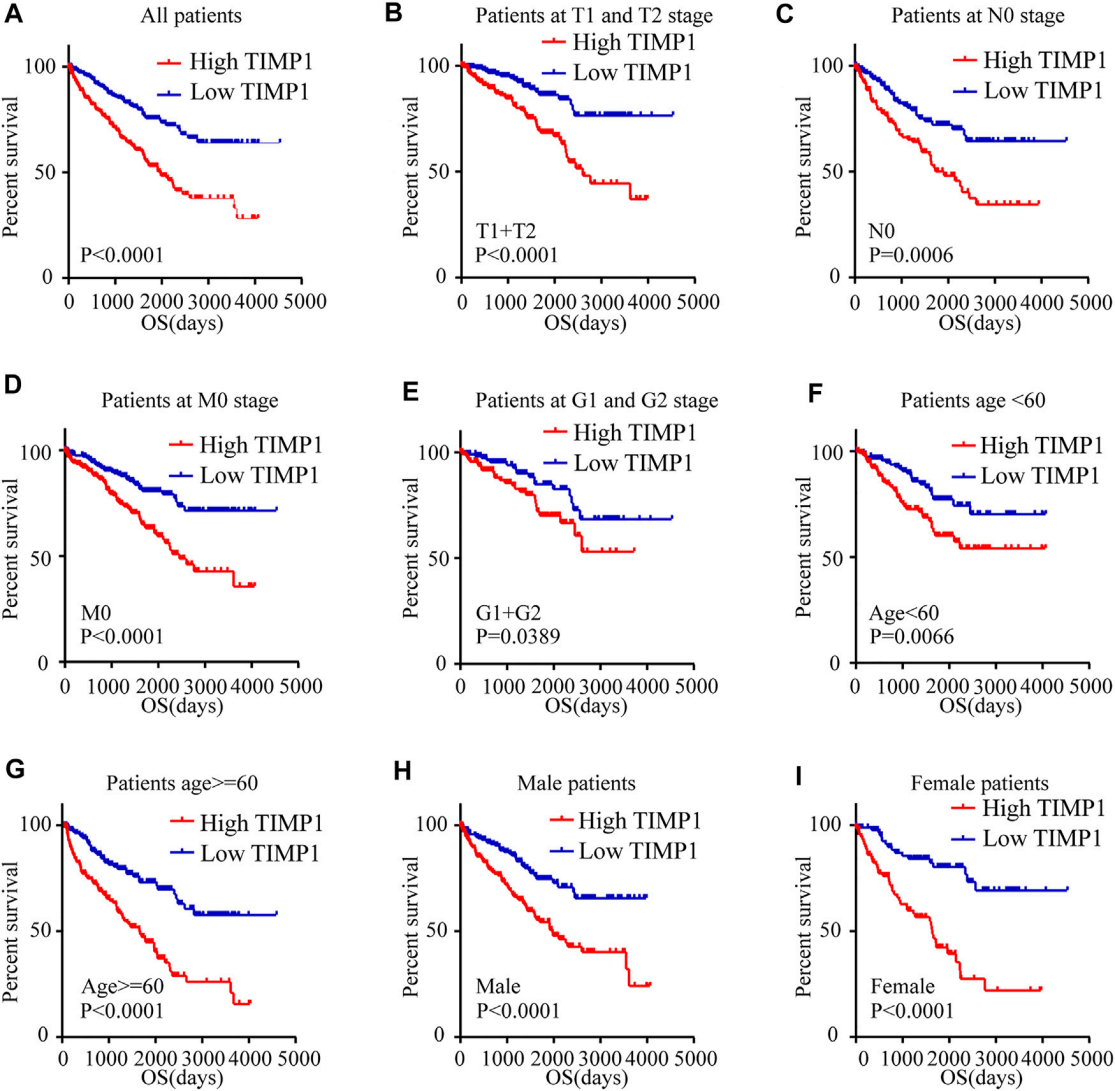
High level of TIMP1 indicated poor OS of patients with RCC. Kaplan-Meier curves for overall survival (OS) were performed in patients with RCC based on the TCGA database. **(A)** OS was closely associated with TIMP1 levels. OS subanalysis was applied in patients with different clinical parameters. **(B)** Patients at T1 and T2 stage, **(C)** Patients at N0 stage, **(D)** Patients at M0 stage, **(E)** Patients at G1 and G2 stage, **(F)** Patient with age<60, **(G)** Patients with age≥60, **(H)** Male patients, **(I)** Female patients. TIMP1, Tissue inhibitor matrix metalloproteinase 1; RCC, renal cell carcinoma; TCGA, The Cancer Genome Atlas.

**FIGURE 4:**
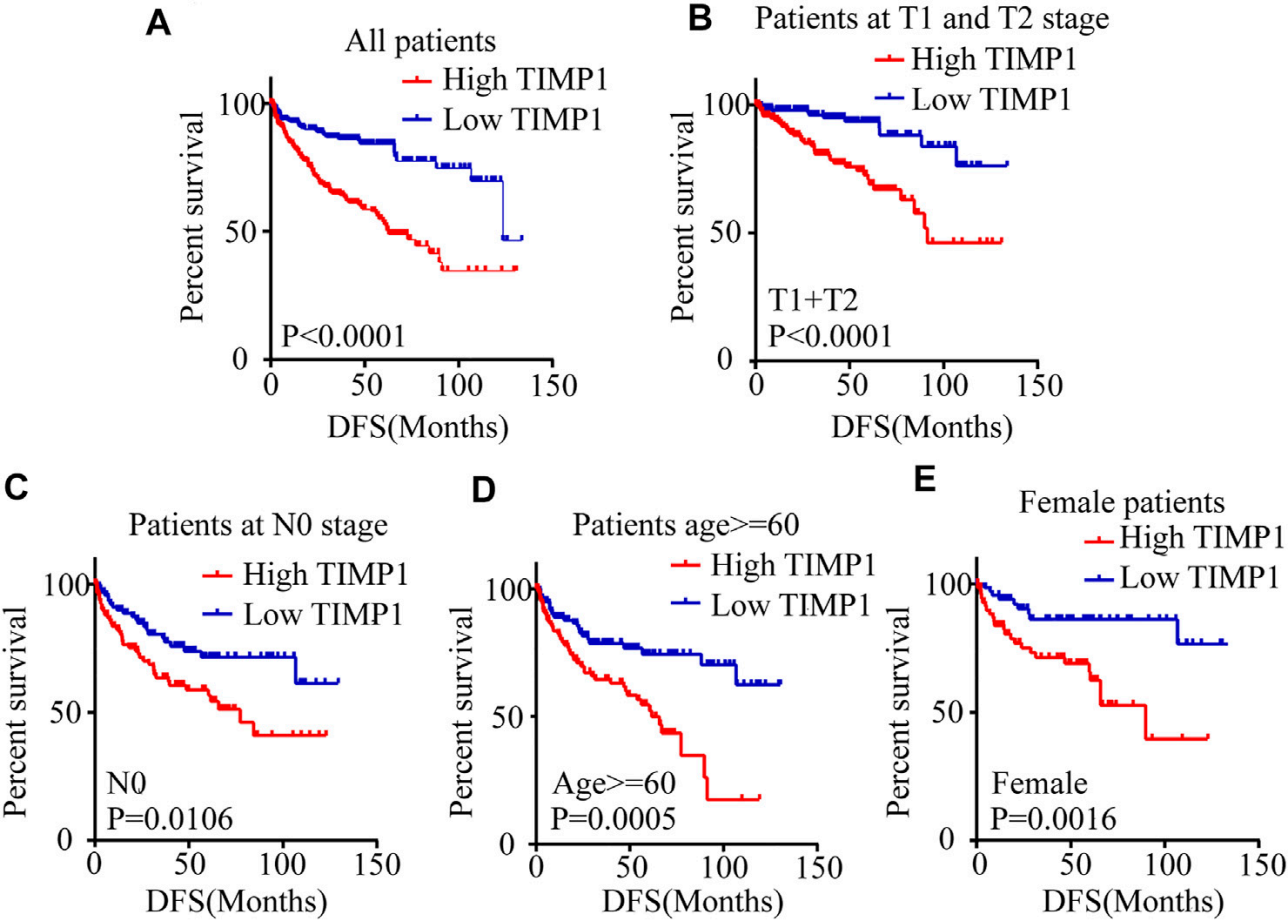
High level of TIMP1 suggested poor DFS of patients with RCC. Kaplan-Meier curves for disease-free survival (DFS) were performed in patients with RCC. **(A)** High level of TIMP1 indicated poor DFS. Kaplan-Meier curves for DFS as determined by different clinical parameters in RCC. **(B)** Patients at T1 and T2 stage, **(C)** Patients at N0 stage, **(D)** Patients with age≥60, **(E)** Female patients. TIMP1, Tissue inhibitor matrix metalloproteinase 1; RCC, renal cell carcinoma; TCGA, The Cancer Genome Atlas.

Kaplan-Meier survival analysis and log-rank test were applied to determine the OS and DFS in patients with RCC. The result showed that patients with higher levels of TIMP1 had a poorer outcome (Figure 3A). Further Kaplan-Meier survival analysis for subgroups of patients with different clinical parameters demonstrated that TIMP1 was an ideal prognostic biomarker for patients with the following characteristics: T1+T2 stage (Figure 3B), N0 (Figure 3C), M0 (Figure 3D), G1+G2 stage (Figure 3E), age<60 (Figure 3F), age≥60 (Figure 3G), male (Figure 3H) and female (Figure 3I). The DFS survival analysis revealed that patients with higher TIMP1 had a shorter disease-free time (Figure 4A), and TIMP1 could act as a biomarker for patients with the following characteristics: T1+T2 (Figure 4B), N0 (Figure 4C), age≥60 (Figure 4D), and female (Figure 4E). Furthermore, we applied univariate and multivariate regression models to assess the integrated prognostic value of TIMP1. The results suggested that TIMP1 independently correlated with the OS and DFS status of RCC patients (Tables 2, 3). In conclusion, the above results demonstrated that the high level of TIMP1 indicated a poor clinical outcome and TIMP1 could serve as an ideal prognostic biomarker for RCC.

**TABLE 2:** Univariate and multivariate analyses of TIMP1 mRNA level and patient overall survival.

Variable	Univariate analysis			Multivariate analysis^a^		
	HR	95% CI	*p*-value	HR^b^	95% CI^c^	*p*-value
Overall survival (n = 522)						
TIMP1	2.375	1.724–3.271	<0.001	1.528	1.080–2.161	0.017
Age (years)	1.786	1.312–2.450	<0.001	1.600	1.159–2.209	0.004
Gender	0.933	0.683–1.275	0.663			
T stage	3.209	2.361–4.364	<0.001	1.572	1.089–2.270	0.016
N stage	3.944	2.135–7.285	<0.001	1.998	1.060–4.136	0.032
M stage	4.351	3.180–5.951	<0.001	2.521	1.766–3.599	<0.001
G grade	2.715	1.925–3.827	<0.001	1.477	1.009–2.162	0.045

a Multivariate models were adjusted for TIMP1, T, N, M, G classification, and age.

b Hazard ratio, estimated from Cox proportional hazard regression model.

c Confidence interval of the estimated HR.

**TABLE 3:** Univariate and multivariate analyses of TIMP1 mRNA level and patient disease-free survival.

Variable	Univariate analysis			Multivariate analysis^a^		
	HR	95% CI	*p*-value	HR^b^	95% CI^c^	*p*-value
DFS (n = 428)						
TIMP1	2.999	2.024–4.442	<0.001	2.104	1.364–3.245	0.001
Age (years)	1.364	0.956–1.946	0.086			
Gender	1.421	0.957–2.112	0.082			
T stage	4.571	3.164–6.603	<0.001	1.954	1.275–2.996	0.002
N stage	6.024	3.024–11.997	<0.001	2.833	1.390–5.774	0.004
M stage	8.522	5.870–12.372	<0.001	4.999	3.317–7.681	<0.001
G grade	3.426	2.269–5.172	<0.001	2.124	1.363–3.309	0.001

a Multivariate models were adjusted for TIMP1, T, N, M, G classification.

b Hazard ratio, estimated from Cox proportional hazard regression model.

c Confidence interval of the estimated HR.

### 3.3 TIMP1 Expression Level Was Associated With Different Clinicopathological Parameters

**FIGURE 5:**
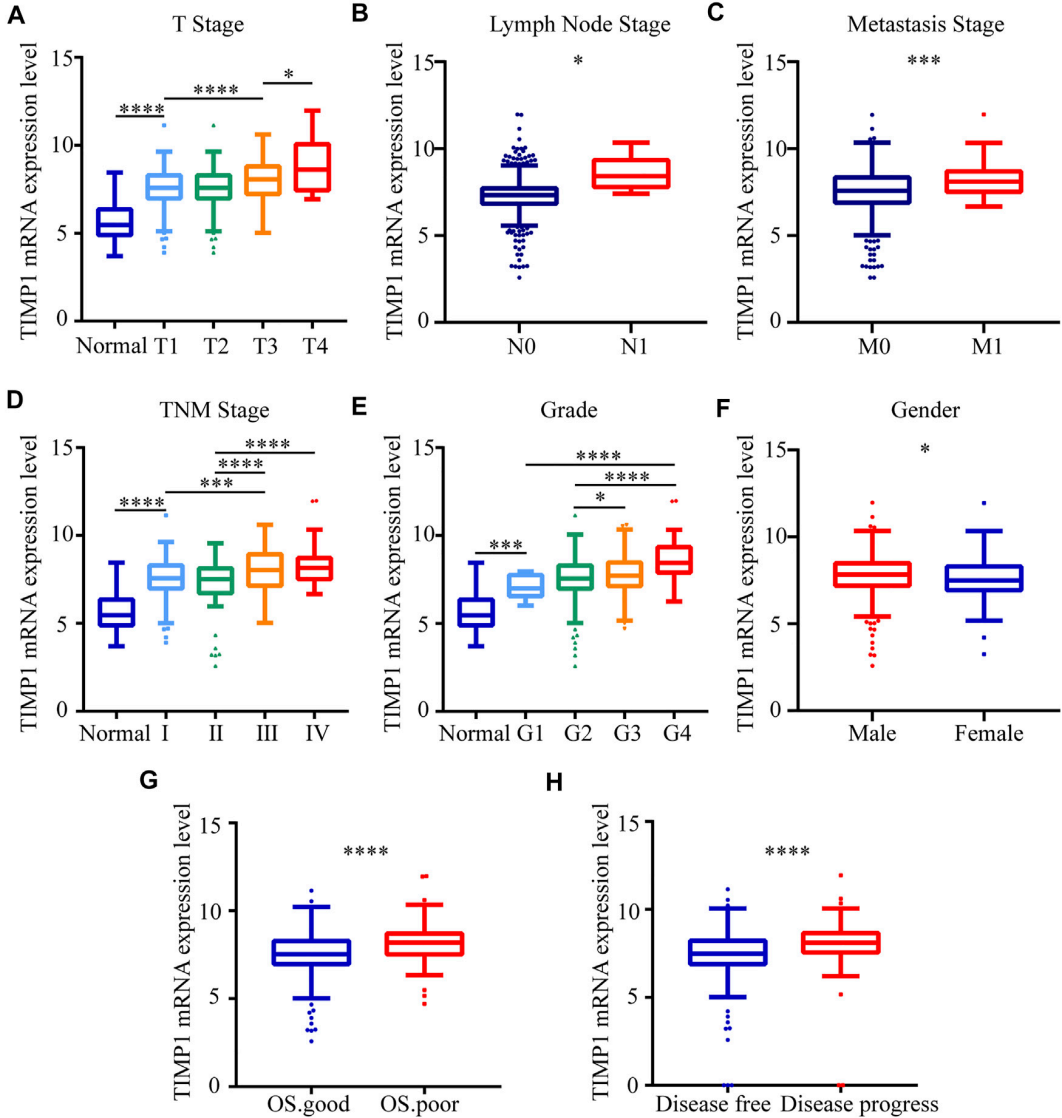
TIMP1 expression level was associated with different clinicopathological parameters. The mRNA expression levels of TIMP1 were downloaded from the TCGA-KIRC database. TIMP1 was upregulated in **(A)** T stage, **(B)** lymph node metastasis, **(C)** distant metastases, **(D)** TNM stage, **(E)** G grade, **(F)** gender, **(G)** OS status, **(H)** DFS status. ****p < 0.0001; ***p < 0.001; *p < 0.05. TIMP1, Tissue inhibitor matrix metalloproteinase 1; RCC, clear cell renal cell carcinoma; KIRC, kidney renal clear cell carcinoma; TCGA, The Cancer Genome Atlas; TNM, Tumor-Node-Metastasis; OS, overall survival; DFS, disease-free survival.

To clarify the expression pattern of TIMP1 in patients with different clinical parameters, the present study analyzed the TIMP1 expression levels of 522 cases from the TCGA database. The results confirmed that the high expression level of TIMP1 is associated with patients’ gender, higher T stage, N stage, M stage, TNM stage, and histological grade (Figures 5A–F). Besides, patients with worse OS status and DFS status had a higher level of TIMP1 expression (Figures 5G,H). However, there were no obvious differences between patients aged ≥60 years and those aged <60 years (Table 4). These results demonstrated that TIMP1 was upregulated and closely related to gender, T stage, N stage, M stage, TNM stage, and histological grade in RCC.

**TABLE 4:** Correlation between TIMP1 mRNA expression and clinicopathological parameters of ccRCC patients.

Parameter		Number	TIMP1 mRNA expression		*p* Value
			Low (*n* = 261)	High (*n* = 261)	
Age (years)	<60	244	119	125	0.599
	≥60	278	142	136	
Gender	Female	180	105	75	0.006^a^
	Male	342	156	186	
T stage	T1 or T2	335	190	145	<0.001^a^
	T3 or T4	187	71	116	
N stage	N0 or NX	507	258	249	0.018^a^
	N1	15	3	12	
M stage	M0 or MX	445	237	208	<0.001^a^
	M1	77	24	53	
G grade	G1 or G2 or Gx	245	146	99	<0.001^a^
	G3 or G4	277	115	162	
TNM stage	I + II	317	186	131	<0.001^a^
	III + IV	205	75	130	

a *p* < 0.05.

### 3.4 The mRNA Expression Level of TIMP1 can Serve as a Biomarker for Clinical RCC Diagnosis

**FIGURE 6:**
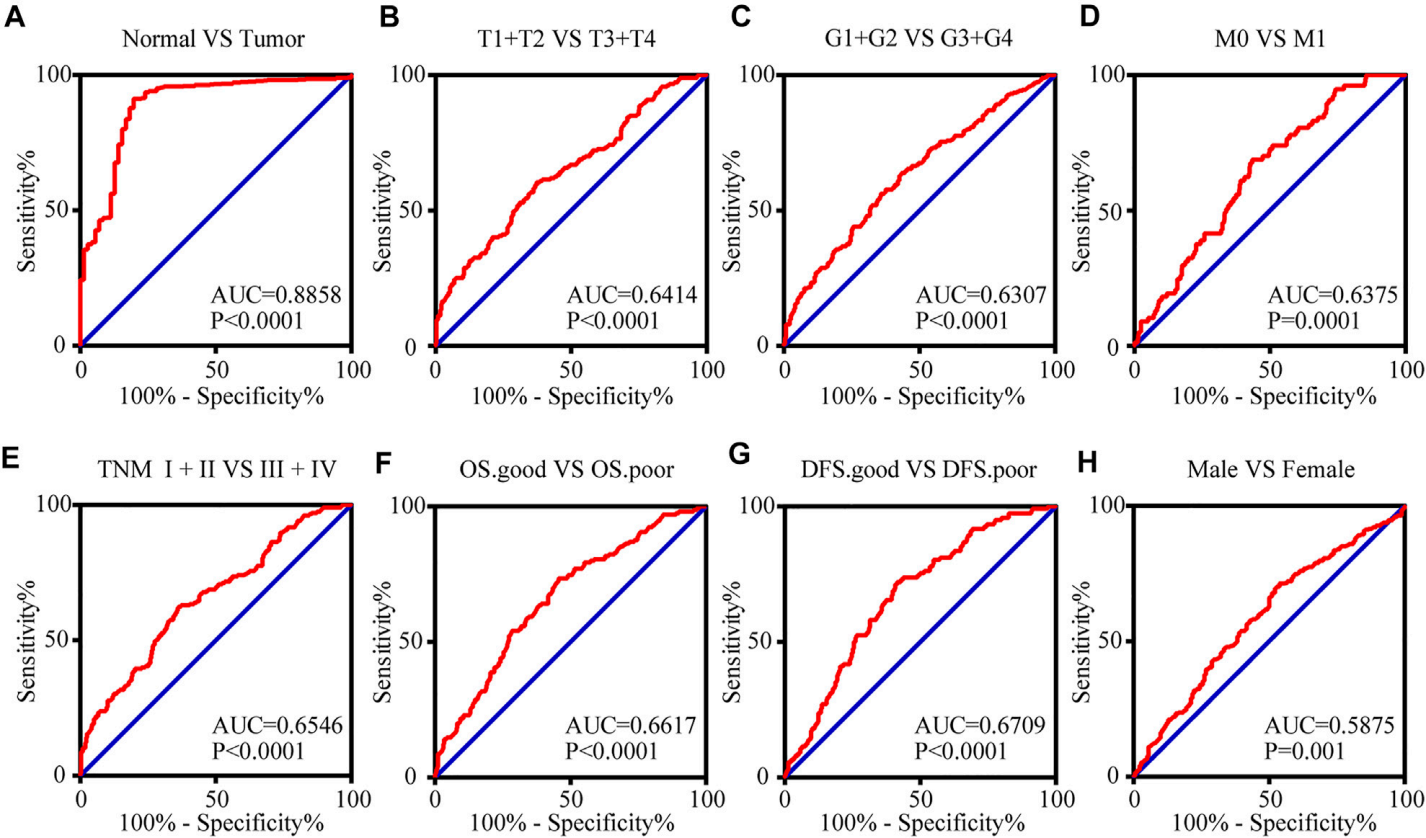
The mRNA expression level of TIMP1 can serve as a biomarker for clinical RCC diagnosis. **(A)** TIMP1 could effectively distinguish RCC from normal tissues (AUC = 0.8858; *p* < 0.0001). ROC analysis was performed in the following subgroups of patients with RCC: **(B)** T grade, **(C)** G stage, **(D)** distant metastases, **(E)** TNM stage, **(F)** OS status, **(G)** DFS status and **(H)** gender. TIMP1, Tissue inhibitor matrix metalloproteinase 1; RCC, clear cell renal cell carcinoma; ROC, Receiver operating curve, AUC, area under the curve; OS, overall survival; DFS, disease-free survival.

To access the value of TIMP1 mRNA expression level in the diagnosis of RCC, receiver operating characteristic (ROC) curves were applied for patients with different clinicopathological variables. Area under curve (AUC) was used to evaluate the diagnostic efficiency. The results indicated that TIMP1 could adequately distinguish RCC patients with an AUC of 0.8858 (*p* < 0.0001; Figure 6A). Additionally, the TIMP1 expression level also exhibited diagnostic value for subgroups of patients with RCC as follows: T1+T2 vs. T3+T4 (AUC = 0.6414, *p* < 0.0001; Figure 6B), G1+G2 vs. G3+G4 (AUC = 0.6307, *p* < 0.0001; Figure 6C), M0 vs. M1 stage (AUC = 0.6375, *p* = 0.0001; Figure 6D), TNM I + II vs TNM III + IV stage (AUC = 0.6546, *p* < 0.0001; Figure 6E), OS good vs. OS poor (AUC = 0.6617, *p* < 0.0001; Figure 6F), DFS good vs DFS poor (AUC = 0.7056, *p* < 0.0001; Figure 6G) and male vs. female (AUC = 0.5875, *p* = 0.001; Figure 6H). Therefore, TIMP1 might act as a potential biomarker for RCC diagnosis.

### 3.5 TIMP1 Was Upregulated in RCC Cell Lines and Tissues

**FIGURE 7:**
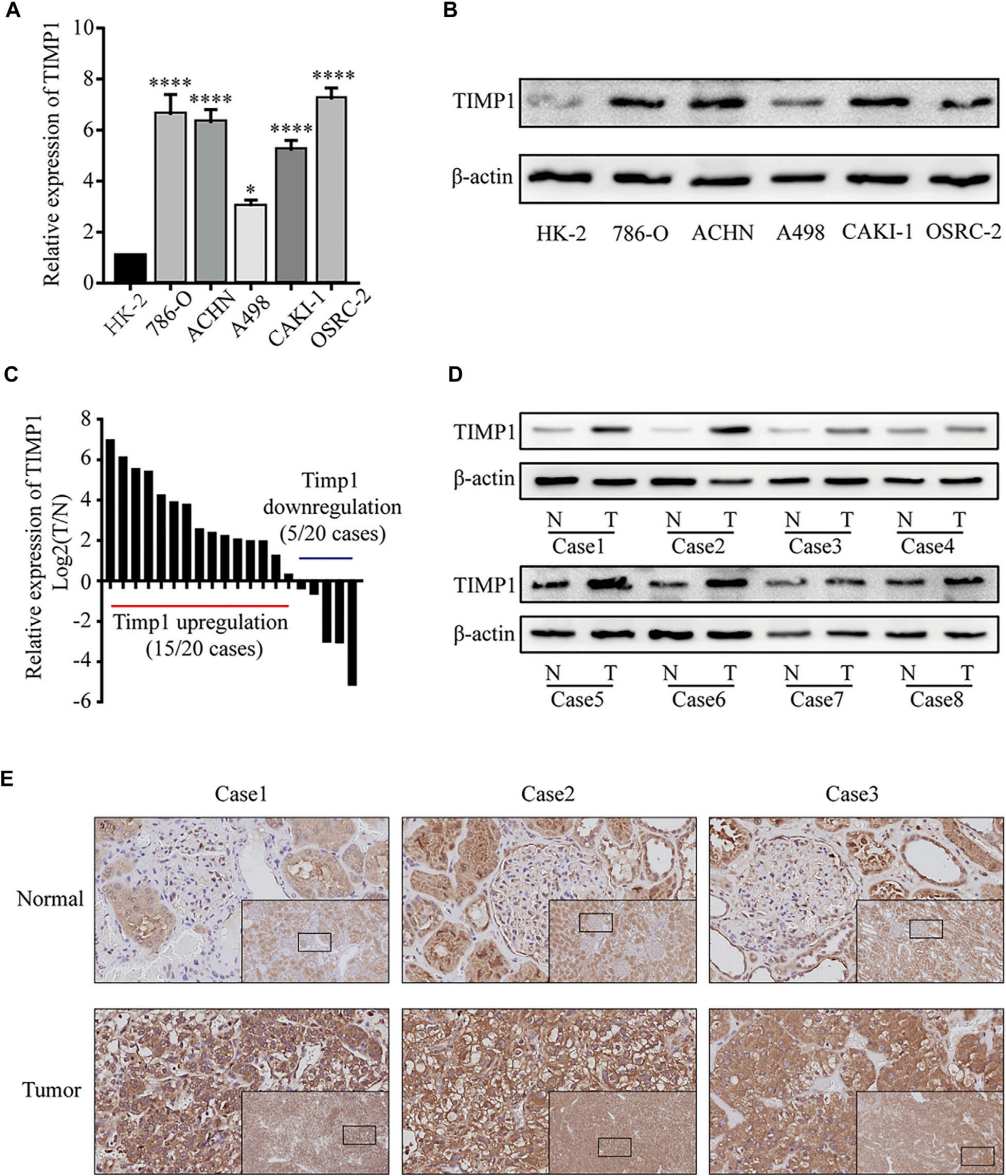
TIMP1 was upregulated in RCC cell lines and tissues. **(A)** Levels of TIMP1 mRNA in 5 renal cancer cell lines (786-O, ACHN, A498, CAKI-1, OSRC-2) and a normal cell line (HK-2). **(B)** Levels of TIMP1 protein in 5 renal cancer cell lines (786-O, ACHN, A498, CAKI-1, OSRC-2) and a normal cell line (HK-2). **(C)** The mRNA levels of TIMP1 in 20 RCC tissues and adjacent nonmalignant tissues. **(D)** The protein levels of TIMP1 in RCC tissues and adjacent nonmalignant tissues. **(E)** Immunohistochemical (IHC) staining for TIMP1 in RCC tissues and adjacent nonmalignant tissues. The images are the lower magnification of the same tissue as that presented in the larger image of each set. Magnification, ×100 and ×400. TIMP1 expression was normalized to β-actin expression. ****p < 0.0001; *p < 0.05. TIMP1, Tissue inhibitor matrix metalloproteinase 1 RCC, clear cell renal cell carcinoma.

To further confirm the results from bioinformatics analysis, we performed a quantitative real-time polymerase chain reaction (qRT-PCR) analysis and western blot in RCC cell lines and tissues. We found a significantly higher level of TIMP1 mRNA in RCC cells (786-O, ACHN, A498, CAKI-1, OSRC-2) relative to HK-2 (Figure 7A). Western blot revealed that the expression level of TIMP1 in RCC was higher compared with HK-2 (Figure 7B). Then we studied the expression of TIMP1 mRNA and protein in tissues. We detected 20 pairs of RCC tissues and corresponding adjacent normal tissues for qRT-PCR and found TIMP1 over-expression in RCC in 17 pairs of tissues (Figure 7C). Western blot for tissues showed a similar result. TIMP1 expression was significantly higher in RCC compared with adjacent normal tissues (Figure 7D). Furthermore, IHC was conducted in 3 pairs of RCC tissues and adjacent normal tissues. TIMP1 was primarily located in the membranes and cytoplasm of cancer cells and renal tubular epithelial cells (Figure 7E). The results proved that the expression of TIMP1 was higher in RCC tissues again. These findings confirmed that TIMP1 was upregulated in RCC cells and tissues.

### 3.6 TIMP1 Promoted the Proliferation, Migration, and Invasion of RCC Cells

**FIGURE 8:**
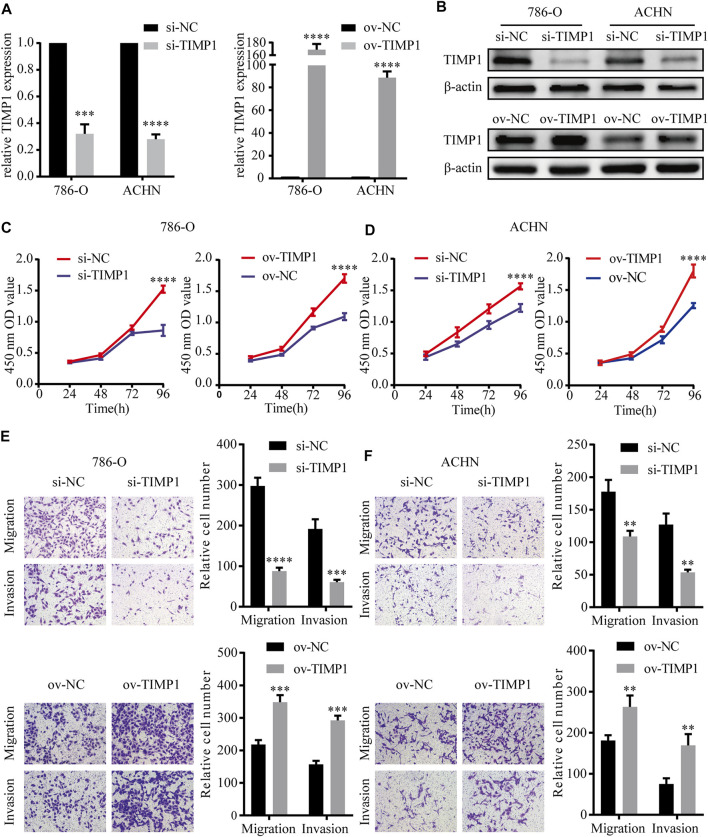
TIMP1 promoted the proliferation, migration and invasion of RCC cells. **(A)** Verification of TIMP1 mRNA levels in transfected 786-O and ACHN cell lines. **(B)** Verification of TIMP1 protein levels in 786-O and ACHN cell lines after knocking down or overexpressing TIMP1. **(C,D)** Cell growth curves of CCK8 assays for transfected 786-O and ACHN cell lines to evaluate cell proliferation. **(E,F)** Transwell assays for transfected ACHN and 786-O cells to evaluate cell migration and invasion ability (Magnification: ×100). All results were plotted as the means ± SEM from three independent experiments. ****p < 0.0001, ***p < 0.001, **p < 0.01. TIMP1, Tissue inhibitor matrix metalloproteinase 1.

**FIGURE 9:**
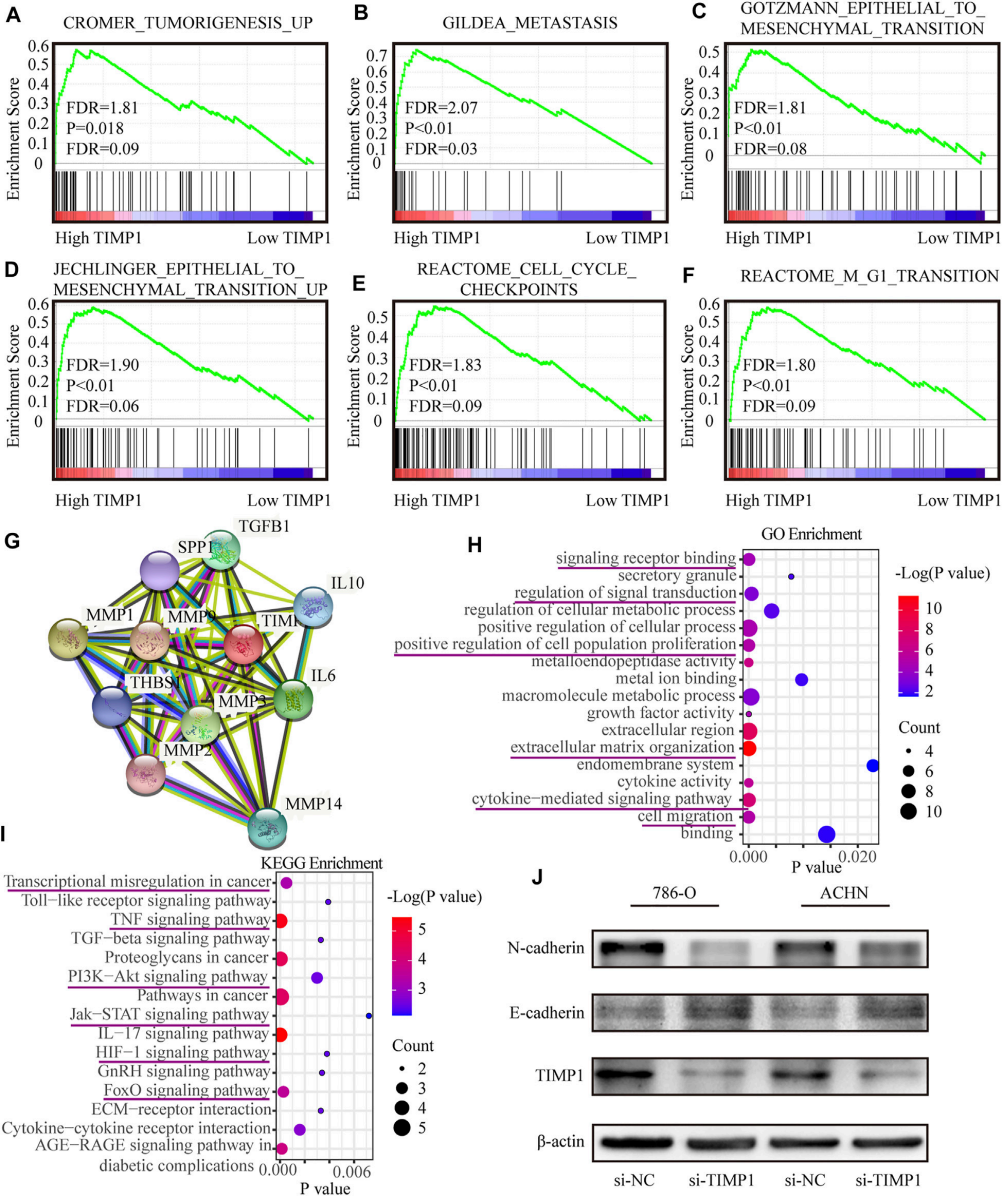
TIMP1 was involved in multiple biological processes and promoted RCC via EMT pathway. **(A–F)** GSEA analysis for the correlations between the biological pathways with the levels of the TIMP1 mRNA based on TCGA database. FDR<25% and *p* < 0.05 were considered statistically significant. **(G)** The protein-protein interaction network of TIMP1. **(H,I)** Biological processes and KEGG pathways in STRING. **(J)** Verification of N-cadherin and E-cadherin protein changes after silencing TIMP1 in 786-O and ACHN. TIMP1, Tissue inhibitor matrix metalloproteinase 1, KEGG (Kyoto Encyclopedia of Genes and Genomes).

In order to elucidate the function of TIMP1 in RCC, TIMP1 was knocked down by TIMP1-siRNA and was overexpressed by transfecting plasmid into ACHN and 786-O cell lines. Then we used qRT-PCR and western blot to test the efficiency of transfection. The results showed that TIMP1-siRNA and plasmid carrying TIMP1 could raise corresponding effects on TIMP1 expression in cells (Figures 8A,B). CCK8 assays revealed that TIMP1 knockdown could significantly repress the proliferation rates of ACHN and 786-O, while overexpression of TIMP1 accelerated cell proliferation (Figures 8C,D). We applied transwell assays to detect the influence of TIMP1 on RCC cells’ migration and invasion ability. The results showed that TIMP1 knockdown suppressed the migration and invasion ability of ACHN and 786-O, while TIMP1 overexpression promoted these features (Figures 9E,F). In conclusion, TIMP1 facilitated the progression of RCC by promoting the proliferation, migration, and invasion of RCC cells.

### 3.7 TIMP1 Was Involved in Multiple Biological Processes and Promoted RCC via the Epithelial-To-Mesenchymal Transition Pathway

To clarify the specific function of TIMP1 in RCC, GSEA based on the TCGA database was performed. Besides, we retrieved the biological process of TIMP1 in STRING (https://string-db.org/). The results of GSEA have shown that TIMP1 was closely related to tumorigenesis, metastasis, cell cycles, and epithelial-to-mesenchymal transition (Figures 9A–F). The protein-protein interaction network calculated by STRING displayed the proteins interacting with TIMP1 (Figure 9G). The major GO term included signaling transduction and cell migration (Figure 9H; Table 5). The KEGG pathways TIMP1 participated in involved PI3K-Akt and JAK-STAT signaling pathways (Figure 9I; Table 6). These results indicated that TIMP1 might play a key role in the metastasis of RCC and participate in extracellular signal transduction. According to the information above, we examined the EMT pathway which was proved to be critical in tumor metastasis by silencing TIMP1. The results revealed that TIMP1 silencing led to the downregulation of N-cadherin and upregulation of E-cadherin (Figure 9J). These findings illustrated that TIMP1 facilitated the progression of RCC *via* EMT transition.

**TABLE 5:** GO enrichment of TIMP1 retrieved from the STRING database.

Term ID	Term description	Observed gene count	Background gene count	FDR	Matching proteins
GO:0030198	Extracellular matrix organization	7	296	4.32E-12	TIMP1, MMP2, TGFB1, THBS1, MMP3, MMP14, MMP1, MMP9, SPP1
GO:0019221	Cytokine-mediated signaling pathway	11	655	5.25E-08	TIMP1, MMP2, TGFB1, MMP3, MMP1, MMP9, IL6, IL10
GO:0016477	Cell migration	7	812	6.15E-06	TGFB1, THBS1, MMP14, MMP1, MMP9, IL6, IL10
GO:0048522	Positive regulation of cellular process	11	4898	1.47E-05	TIMP1, MMP2, TGFB1, THBS1, MMP3, MMP14, MMP1, MMP9, SPP1, IL6, IL10
GO:0008284	Positive regulation of cell population proliferation	8	878	7.74E-06	TIMP1, MMP2, TGFB1, THBS1, MMP9, IL6, IL10
GO:0043170	Macromolecule metabolic process	9	7453	0.00039	TIMP1, MMP2, TGFB1, THBS1, MMP3, MMP14, MMP1, MMP9, SPP1, IL6, IL10
GO:0009966	Regulation of signal transduction	11	3033	0.00052	TIMP1, TGFB1, THBS1, MMP14, MMP9, SPP1, IL6, IL10
GO:0031323	Regulation of cellular metabolic process	8	6082	0.0042	TIMP1, TGFB1, THBS1, MMP3, MMP14, MMP9, SPP1, IL6, IL10
GO:0005576	Extracellular region	9	2505	4.08E-09	TIMP1, MMP2, TGFB1, THBS1, MMP3, MMP14, MMP1, MMP9, SPP1, IL6, IL10
GO:0012505	Endomembrane system	11	4347	0.0228	TIMP1, TGFB1, THBS1, MMP14, MMP9, SPP1, IL6
GO:0004222	Metalloendopeptidase activity	7	110	2.39E-07	MMP2, MMP3, MMP14, MMP1, MMP9
GO:0005125	Cytokine activity	5	216	1.31E-05	TIMP1, TGFB1, SPP1, IL6, IL10
GO:0008083	Growth factor activity	4	160	1.77E-05	TIMP1, TGFB1, IL6, IL10
GO:0046872	Metal ion binding	7	4087	0.0097	TIMP1, MMP2, THBS1, MMP3, MMP14, MMP1, MMP9
GO:0005488	Binding	11	11878	0.0143	TIMP1, MMP2, TGFB1, THBS1, MMP3, MMP14, MMP1, MMP9, SPP1, IL6, IL10
GO:0030141	Secretory granule	4	828	0.0078	TIMP1, TGFB1, THBS1, MMP9
GO:0012505	Endomembrane system	7	4347	0.0228	TIMP1, TGFB1, THBS1, MMP14, MMP9, SPP1, IL6

GO, gene ontology; TIMP1, Tissue inhibitor matrix metalloproteinase 1; FDR, false discovery rate.

**TABLE 6:** KEGG enrichment of TIMP1 retrieved from the STRING database.

Term ID	Term description	Observed gene count	Background gene count	FDR	Matching proteins
hsa04657	IL-17 signaling pathway	4	92	3.45E-06	MMP3, MMP1, MMP9, IL6
hsa04668	TNF signaling pathway	4	108	5.13E-06	MMP3, MMP14, MMP9, IL6
hsa05205	Proteoglycans in cancer	4	195	3.19E-05	MMP2, TGFB1, THBS1, MMP9
hsa05200	Pathways in cancer	5	515	4.61E-05	MMP2, TGFB1, MMP1, MMP9, IL6
hsa04068	Fofo signaling pathway	3	130	0.00027	TGFB1, IL6, IL10
hsa05202	Transcriptional misregulation in cancer	3	169	0.00052	MMP3, MMP9, IL6
hsa04060	Cytokine-cytokine receptor interaction	3	263	0.0016	TGFB1, IL6, IL10
hsa04151	PI3K-Act signaling pathway	3	348	0.003	THBS1, SPP1, IL6
hsa04350	TGF-beta signaling pathway	2	83	0.0033	TGFB1, THBS1
hsa04512	ECM-receptor interaction	2	81	0.0033	THBS1, SPP1
hsa04912	GnRH signaling pathway	2	88	0.0034	MMP2, MMP14
hsa04066	HIF-1 signaling pathway	2	98	0.0038	TIMP1, IL6
hsa04620	Toll-like receptor signaling pathway	2	102	0.0039	SPP1, IL6
hsa04630	Jak-STAT signaling pathway	2	160	0.0072	IL6, IL10
hsa04933	AGE-RAGE signaling pathway in diabetic complications	3	98	0.00014	MMP2, TGFB1, IL6

KEGG, kyoto encyclopedia of genes and genomes; TIMP1, Tissue inhibitor matrix metalloproteinase 1; FDR, false discovery rate.

## 4 Discussion

RCC is one of the most common malignant tumors in the urinary system and accounts for 1.8% of deaths from cancers (Bray et al., 2018; Wan et al., 2019a; Wan et al., 2019b; Luo et al., 2019; Xu et al., 2019; Zhang et al., 2019; Zhou et al., 2019). So far, there is no effective systemic therapy. Due to the lack of effective tumor markers for early screening and the mild symptoms of early-stage RCC, 1/3 patients have ectopic metastases when diagnosed (Gong et al., 2016). For patients with distant metastases, first-line treatments mainly include surgical resections and TKI inhibitors such as sunitinib, pazopanib, and axitinib (Rini et al., 2011). There are also phase 3 clinical trials showing that everolimus (a mTOR inhibitor), compared with placebo, has a longer disease-free survival (Choueiri et al., 2015; Motzer et al., 2015; Cella et al., 2016). However, the development of drug resistance leads to their failure and brings some adverse reactions such as liver toxicity, hand-foot syndrome, etc (Motzer et al., 2013). Thus, detecting RCC at early stage is of most importance for improving patients’ survival by far.

Some most recent researches showed RAC2, LINC00160, IGFL2-AS1, AC023043.1 could serve as biomarkers for diagnosing RCC (Cheng et al., 2019; Liu et al., 2019; Cheng et al., 2020). In the present study, we selected the members of the TIMP family, which encoded the natural inhibitors for MMPs. We found an independent prognostic factor for RCC, TIMP1, by using bioinformatics analysis. We discovered that TIMP1 was significantly up-regulated in RCC and patients with a higher level of TIMP1 had worse clinical outcomes. ROC analysis revealed that TIMP1 could distinguish RCC patients from normal people. Meanwhile, TIMP1, as a secreted protein, could be detected in blood and other body fluid. So TIMP1 might be an ideal biomarker for RCC according to these findings.

Related research showed that TIMP1 could inhibit the proteolytic activity of matrix metalloproteinases (MMPs) by forming noncovalent 1:1 stoichiometric complex and regulate the balance of matrix remodeling during degradation of extracellular matrix (Batra et al., 2012). However, the most recent studies revealed other important biological functions of TIMP1 including anti-apoptosis, anti-angiogenesis, cell cycle regulation, and differentiation activities. TIMP1 activated hepatic stellate cells via CD63 signaling to create a premetastatic niche in pancreatic cancer (Grunwald et al., 2016). Down-regulation of TIMP1 was found to enhance gemcitabine sensitivity and reverse chemoresistance in pancreatic cancer (Tan et al., 2020). TIMP1 was involved in angiogenesis in gastric cancer (Li et al., 2020). TIMP1 could even regulate the adipogenesis of adipose-derived stem cells via the WNT signaling pathway (Wang et al., 2020). Besides, TIMP1 was associated with fibrosis and suppression of programmed cell death of B Cells (Guedez et al., 1998; Arthur, 2000; LaRocca et al., 2017). The upregulation of TIMP1 was relative to poor prognosis of multiple cancers including colon cancer, breast cancer, gastric cancer, melanoma, papillary thyroid carcinoma, renal cell carcinoma, and so on (Hawthorn et al., 2004; Wang et al., 2013; Cheng et al., 2016; Song et al., 2016; Zurac et al., 2016).

To further investigate the function of TIMP1 in RCC, we applied qRT-PCR, WB, and IHC to confirm the upregulation of TIMP1 in RCC cells and tissues. Next, we researched the effect of TIMP1 on RCC cells by knocking down and overexpressing TIMP1. The results revealed that TIMP1 promoted proliferation, migration, and invasion of RCC cells. In order to figure out how TIMP1 facilitated RCC progression, we applied GSEA based on TCGA_KIRC and retrieved the biological functions of TIMP1 via STRING. The discovery showed that TIMP1 mainly participated in the regulation of extracellular matrix and closely associate with metastasis, EMT pathway, and some typical signal transduction pathways. Further study indicated that knockdown of TIMP1 led to up-regulation of E-cadherin and down-regulation of N-cadherin, which proved TIMP1 accelerated the progression of RCC via EMT pathway in a MMPs inhibitor-independent manner.

The EMT signal pathway was well known for its critical function in wound healing, tumor metastasis and malignant progression (Dongre and Weinberg, 2019; Brabletz et al., 2021). The cells developed into a quasi-mesenchymal state from the original epithelial state via the EMT pathway, which strengthens the metastatic potential of malignant cells (Kalluri and Weinberg, 2009; Nieto, 2009; Thiery et al., 2009; Nieto et al., 2016). Recent studies showed that TIMPs and MMPs were closely related to the EMT pathway, which supported our findings (Argote Camacho et al., 2021; Nayim et al., 2021). But studies involving the specific mutual effect between TIMP1 and the EMT pathway were rare.

Taking the above findings into consideration, it was reasonable to believe that the most critical function of TIMP1 was to enhance the metastatic ability of RCC cells via the EMT pathway, while the effect of TIMP1 on proliferation might be due to other minor functions of the EMT pathway. All these discoveries showed TIMP1 might be a potential diagnostic and prognostic biomarker for clear cell renal cell carcinoma that facilitated tumor progression. There were still some limitations in the present study. The effect of TIMP1 was not proved *in vivo*. Besides, the specific mechanism causing the abnormal regulation of TIMP1 was still unclear. Therefore, further study was required to solve these problems.

## 5 Conclusion

The present research proved that a high level of TIMP1 expression was associated with a poor clinical outcome. TIMP1 promoted the proliferation, migration, and invasion of RCC cells and facilitated the progression of RCC *via* the EMT pathway. We proved that the biological effects of TIMP1 mediated by signal transduction pathways were far more than those previously known as MMP inhibitors. The aforementioned results indicated that TIMP1 may be an ideal diagnostic and prognostic biomarker for RCC, and molecular targets for TIMP1 might provide a new choice for RCC treatment.

## Data Availability

The datasets presented in this study can be found in online repositories. The names of the repository/repositories and accession number(s) can be found in the article/Supplementary Material.
