# Supplementary figures and images for: TIMP1 Indicates Poor Prognosis of Renal Cell Carcinoma and Accelerates Tumorigenesis via EMT Signaling Pathway
Source: Front Genet. 2022 Feb 25;13:648134. doi: 10.3389/fgene.2022.648134 (PMC8914045; doi:10.3389/fgene.2022.648134)

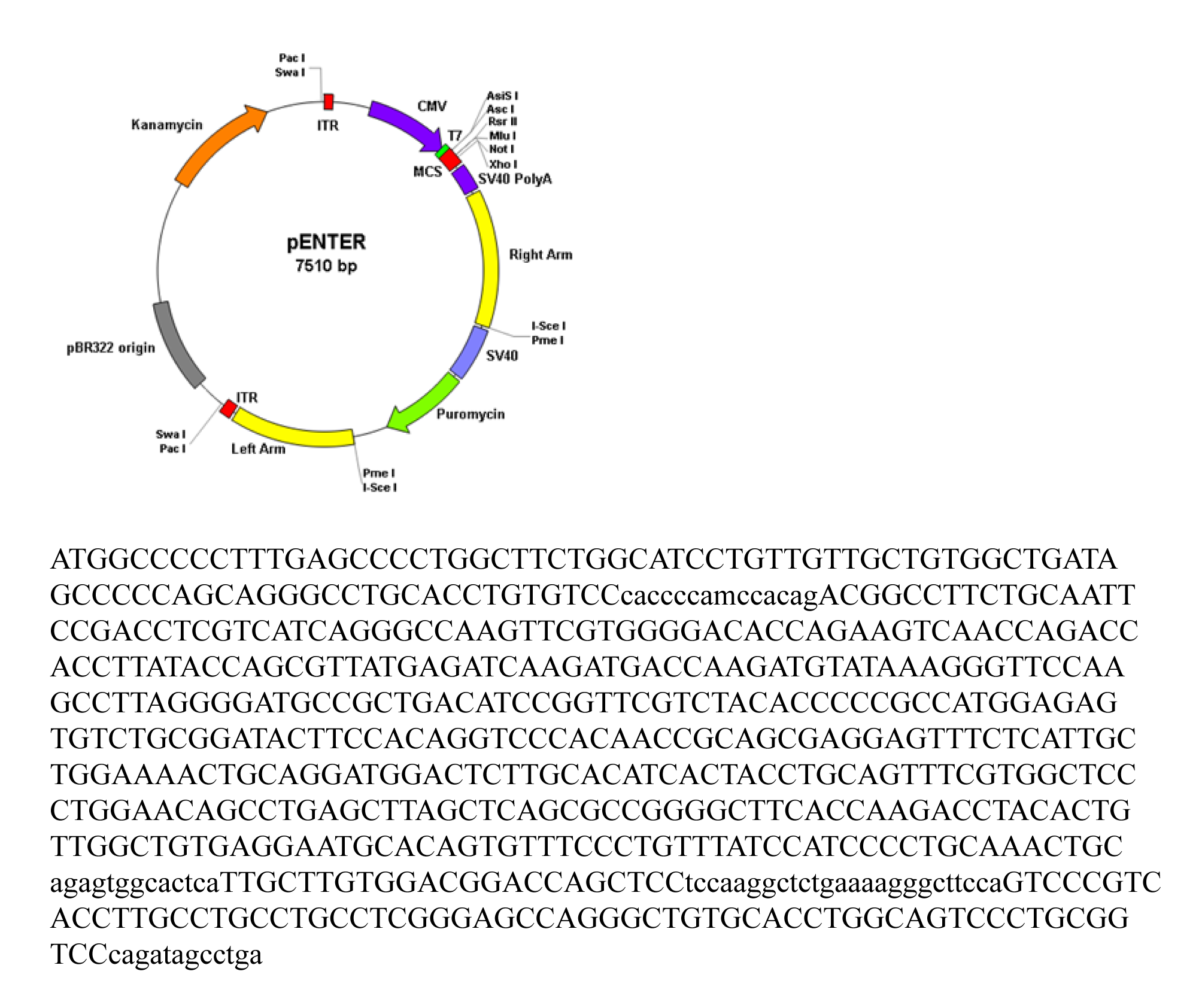

Supplement: Supplementary file 2 [file Image1.TIF]
